# A protocol for social interactive assessment of infant attention set-shifting between 12–24 months of age

**DOI:** 10.1016/j.mex.2023.102273

**Published:** 2023-06-29

**Authors:** Xing Xi Tan, Victoria Leong

**Affiliations:** a Division of Psychology, Nanyang Technological University, Singapore; b Department of Psychology, University of Cambridge, Cambridge, UK

**Keywords:** Switching, Set-shifting, Cognitive flexibility, Executive function, Parent-Child Interaction, Attentional Set-shifting Sequential Touching Task

## Abstract

This protocol describes an adaptation of a classic sequential touching object categorisation task to assess infant attention set-shifting, suitable for ages 12–24 months. The task is conducted in a social interactive context with a parent, who scaffolds their infants’ attention shift from high-salience to low-salience dimensional properties of objects (e.g., shape vs material). This task is adapted from Ellis and Oakes (2006), where 14 month-old infants were able to flexibly attend to both shape and material. In this paper, we present a methodological innovation which permits the direct measurement of the effect of parent-child interactions on an early developing executive function skill. This novel social interactive protocol permits direct assessment of the effect of parent-child interaction on an early executive function skill, attention set-shifting.

• The parental role is to scaffold a shift in their child's attention from a high salient (e.g. shape) to a low-salient (e.g. material) dimension of the stimulus set.
• The protocol is suitable for infants aged between 12 and 24 months.

## Graphical abstract

**Figure:**
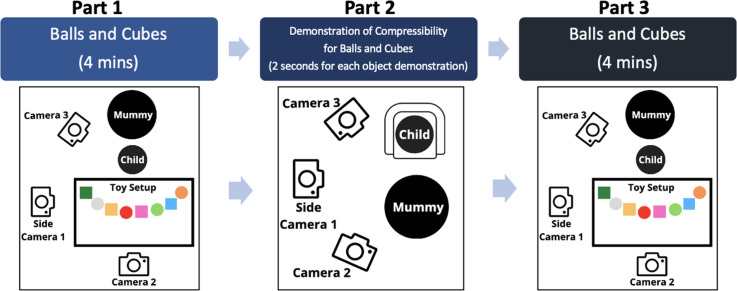

Specifications table

**Table:** 

Subject area:	Psychology
More specific subject area:	Cognitive Development in Infants and Children
Name of your method:	Attentional Set-shifting Sequential Touching Task
Name and reference of original method:	The protocol was adapted from the task methodology from Ellis and Oakes' paper: Flexible categorization in infants were investigated using a set of objects that can be categorized by shape (balls vs. blocks) or material (soft vs. hard)
	Ellis, A. E., & Oakes, L. M. [1]. Infants flexibly use different dimensions to categorize objects. *Developmental psychology*, 42(6), 1000–1011. 10.1037/0012–1649.42.6.1000
Resource availability:	NA

## Method details

### Aims and rationale

Parent-child interactions are known to be an important factor in early developing cognition, and for executive function (EF) in particular [4,5]. However, most EF tasks do not permit a direct assessment of the effect of parental interactions on EF performance in children. Studies that have examined such social influences generally use a correlational design rather than measuring parental interactions in situ during EF task performance. Consequently, there exists a gap in understanding how real-time parent-child interactions may help to scaffold EF development, particularly in young children. Here, we present a new Interactive Executive Function (EF) task for assessment of infant attention set-shifting in a social interactive context. This is intended to provide reliable psychometric measurement of early EF precursors, and may be used as an observational tool or developmental assessment. We expect the task to provide deep insights at a microanalytic level into the social interactive behaviour between parent and child. And for further insights on how these directional social influences (e.g., the dynamic exchange of parental ostensive signals and infants’ responses to these signals) may influence attentional shifts by the infant during performance of the task. In a lab environment, task administration can be executed with a high degree of experimental control. However, this might not be achievable or desirable in the home context. Accordingly, the current task was adapted in a manner to ensure low task demands and simplified instructions, so that the task can also be easily administered in a naturalistic home context. This protocol paper describes a novel Interactive EF task protocol for reliable psychometric measurement of an early EF precursor, attention set-shifting, and which directly incorporates parental social interaction as an experimental factor. This novel interactive measure is adapted from reliable and valid EF tasks used with older children [2,3] and designed to be age-appropriate for infants aged between 12 and 24 months, and permit reliable parental involvement in the task administration.

**Fig. 1:**
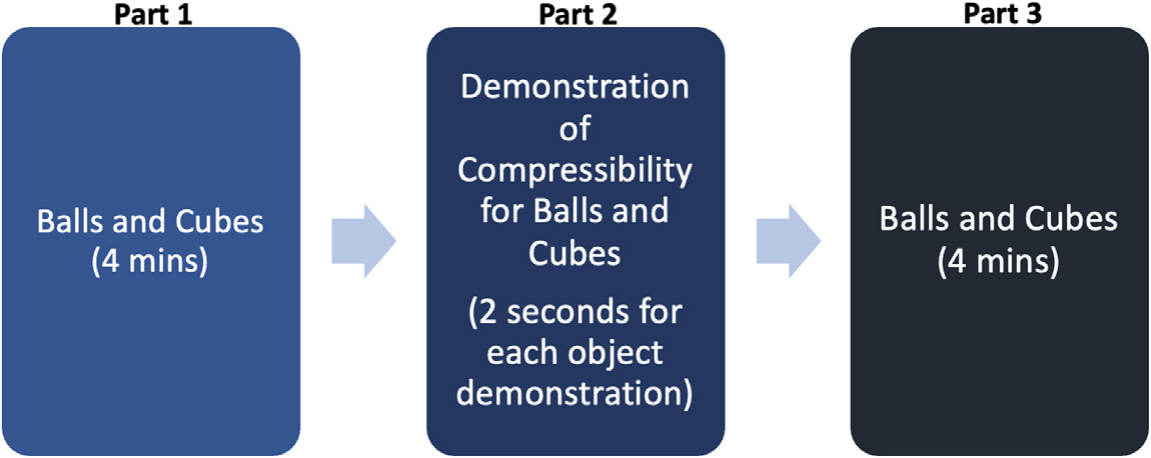
Overall Task Flow.

This task comprises of three parts, conducted over a duration of 20 min (including setup time), as illustrated in Fig. 1:

• Part 1: Pre-demonstration free play (4 min)
• Part 2: Material (compressibility) demonstration by parent
• Part 3: Post-demonstration free play (4 min)

For Parts 1 and 3, 8 different objects are placed in front of the infant, and s/he is allowed to interact freely with them. The objects are placed in a semi-circle formation to maintain the same distance between each toy and the infant. The objects are selected so that they differ systematically according to shape (4 balls vs. 4 blocks) or material (4 soft vs. 4 hard), and therefore include a combination of hard and soft toy blocks, as well as hard and soft toy balls. The size of each object is standardised, but their colour and pattern are randomised to maintain infants’ interest in the toys. Infants’ sequence of touches across the different toys is noted and scored (Table 1). Next, during Part 2, the parent draws their infants’ attention to a different dimension of the toys – the compressibility of the material. Parent will demonstrate this by squeezing or compressing each toy using their fingers in view of their infant. After this demonstration, the infant is again allowed to interact freely with the objects. Sociometric coding of infant and parental behaviour (e.g., for gaze and type/quality of play) is then applied to the video data as summarised in Table 1.

**Table 1:** Sociometric coding of infant and parental behaviour for STT task.

Brief Video Coding Legend
80.x: Infant Vocalisations
81.x: Infant Facial Expressions
Part 1: Pre-demonstration free play
20: Code for entire duration of free play
21.x-25.x: Infant's intentional touch/gaze, functional/non-functional play with toys
Part 2: Material (compressibility) demonstration by parent
90: Duration of parental demonstration of compressibility
91.x-95.x: Infant's Gaze and Touch on each object during demonstration
Part 3: Post-demonstration free play
30: Code for entire duration of free play
31.x-35.x: Infant's intentional touch/gaze, functional/non-functional play with toys

### Participants

This task measure is suitable for infants aged between 12 months to 24 months and require the participation of one parent or adult caregiver.

### Materials

(1) Standardised Toys (4 Balls and 4 Cubes)

**Table:** 

Balls (2 compressible, 2 rigid)		Cubes (2 compressible, 2 rigid)	
Hard Material	Soft Material	Hard Material	Soft Material
- 1 White and Red ball, (approximately 6 cm in diameter)	- 1 Orange ball (approximately 6 cm in diameter)	- 1 Green cube (5 x 5 x 5 cm)	- 1 Pink cube (5 x 5 x 5 cm)
- 1 White and Black ball, (approximately 6 cm in diameter)	- 1 Green ball (approximately 6 cm in diameter)	- 1 Brown cube (5 x 5 x 5 cm)	- 1 Light Blue cube (5 x 5 x 5 cm)

(2) Presentation tray (54 x 79 x 12 cm corrugated board with raised sides)
(3) Video Cameras
  • Camera 1: Side view camera
  • Camera 2: Front view camera
  • Camera 3: Back view camera
(4) 1 pair of Eye-tracking glasses for Mother
(5) 1 Stopwatch
(6) 1 Infant high chair
(7) Experimenter Documents
  a. Session sheet and Experimenter Checklist (Supplementary Material Appendix 1)
  b. Toy order (Supplementary Material Appendix 2)
  c. Parent Instruction sheet (Supplementary Material Appendix 3)

### Staff personnel

This task optimally requires two lab staff (experimenters), one technical support and the mother who will administer the task. The recommended roles are:

Experimenter 1 (E1)

• Remains in the room throughout the task
• Briefs the mother on instructions
• Uses the stopwatch to signal to the mum when to move to the next part
• Moves the equipment (i.e., video cameras, play setup) in between each part

Experimenter 2 (E2, optional)

• Plays with the infant when E1 is briefing the mother
• Assists with preparation
• Leaves the room during the task

Technical Support

• Ensure good functioning of all equipment
• Moves the equipment (i.e., video cameras, play setup) in between each part

### Sociometric sensor equipment

The collection of sociometric data requires a set of audio-visual equipment of recommended specifications optimised for high quality video and audio recording. Table 2 lists recommended models of video cameras, audio microphones, and eye trackers.

**Table 2:** List of Sociometric Sensor Equipment.

Item	Number of sets	Specifications
Video Cameras SONY FDR-AX700 4 K HDR Camcorder	3	- Video Resolution: [PAL] XAVC S 4K: 3840 x 2160/25p - Video Recording Rate (ABR/VBR): XAVC S 4K: Approx. 60 Mbps - Effective Pixels: Approx. 14.2 M pixels (16:9)
Audio Recorder Wireless GO II (Dual Channel Wireless Microphone System)	1 (1 receiver and 2 transmitters – 1 for Mother, 1 for Infant)	- Dual channel wireless microphone system for recording two sound sources simultaneously - Series IV 2.4 GHz digital transmission, 128-bit encryption
Eye Tracker Glasses Tobii Pro-Glasses 3	1 (for Mother)	- Scene camera with a wide field of view (106° H: 95°, V: 63°)

Sociometric coding of infant and parental behaviour and eye gaze is applied to all 3 parts of the task. Infant and parental behaviour are captured using video cameras angled at strategic locations around the testing room to capture movements of the hands, arms and upper torso. Eye gaze and eye contact between parent and infant is captured using Tobii Pro-Glasses 3 worn by the parent.

## Task procedure

### Parental role and briefing

Before the start of the task, the mother is to be briefed about the instructions of the task (see Appendix 3 for detailed instructions). During the briefing, mothers are to be taught the way to compress the toys during demonstration. The compression practice involves 1 soft orange ball and 1 soft blue cube that is standardized across all participants. When learning to compress the toys, ensure that mothers learn to compress and hold the toy for 2s. Additionally, during the briefing, ensure that the infant does not have access to the materials used in the experiment and cannot see the experimenter or the mother compressing the objects. During the experiment, the role of the parent is to lead their child in performing the task, providing appropriate demonstrations and prompts to elicit the required responses. The parent's role is especially crucial during the Demonstration phase (Part 2) to scaffold their infants’ attentional shift to the low-salient dimension of the objects (compressibility). Parents are supported by experimenters who assist to monitor task administration. During Part 1, parents are given instructions to be seated behind their infants and provide a verbal cue (e.g., Let's play with these toys) to encourage their infant to interact with the objects placed before them. After providing this brief verbal cue, parents are to remain quiet and allow their infants to play with the toys. During Part 2, parents are instructed to position their infant in a high-chair and secure his/her attention before demonstrating the compressibility of each object. In Part 3, parents are given the same instructions as in Part 1. Throughout the task, the experimenter will interact minimally with the parent-infant dyad. The experimenter will be stationed behind a video camera to record the task and monitor the task administration.

### Experimental setup

**Fig. 2:**
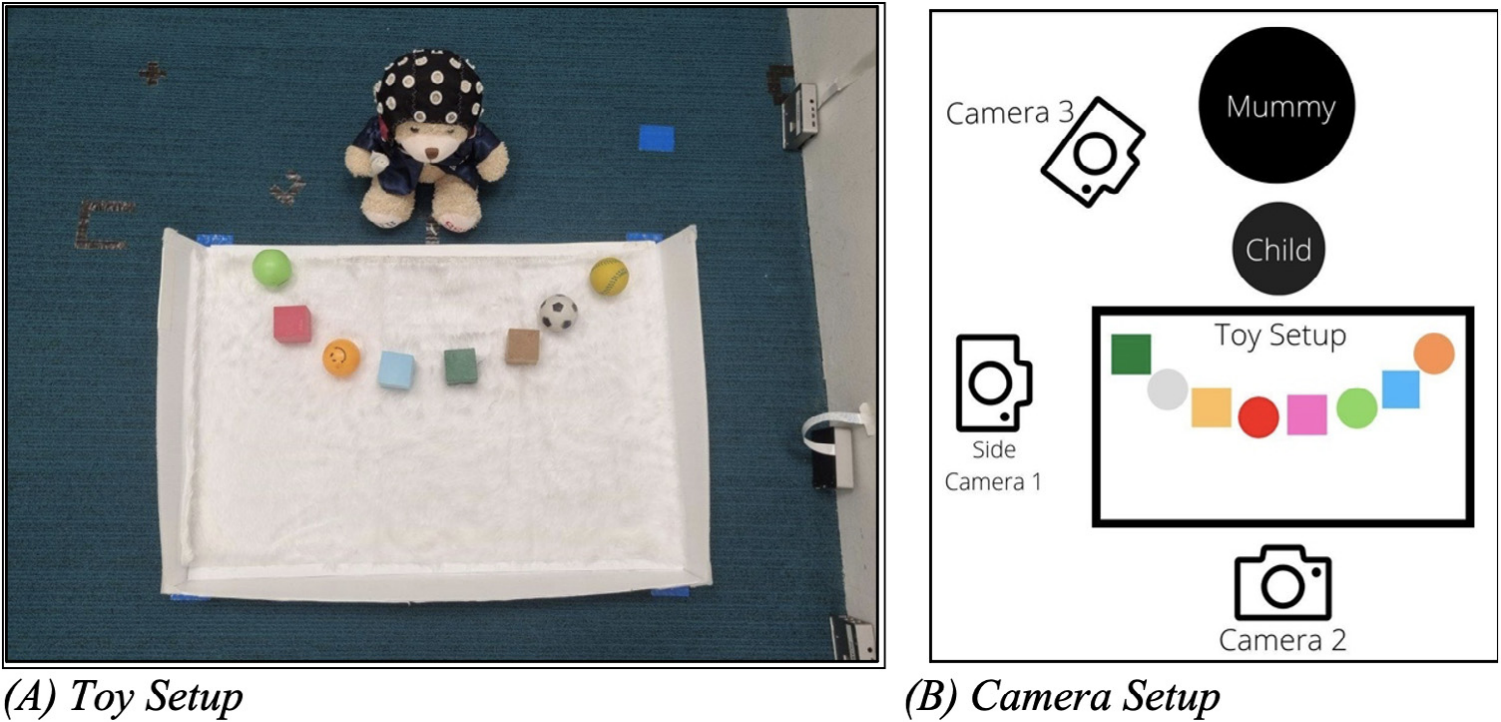
(A) Toy Setup, (B) Camera Setup.

The infant is to be seated on the floor for Parts 1 and 3 (ensure that the room is clear and safe). The order in which the objects are placed on the tray is predetermined. There are three possible orders: 1/2/3 (see Appendix 2). Experimenters are to set up the presentation tray on the floor and lay out the balls and cubes on the tray according to the chosen toy order (see Fig. 2A, infant is depicted using the teddy bear). Camera positions are to capture mainly the play area, infant's torso and face, and mother's face (see Fig. 2B for an example of Camera setup). Camera 1 (Side view camera) to capture the side view of play area. Camera 2 (Front view camera) to capture the infant's torso and eyes, mother's face, and play area. Camera 3 (Back view camera) to capture infant's back torso view, mother's head, and play area.

**Fig. 3:**
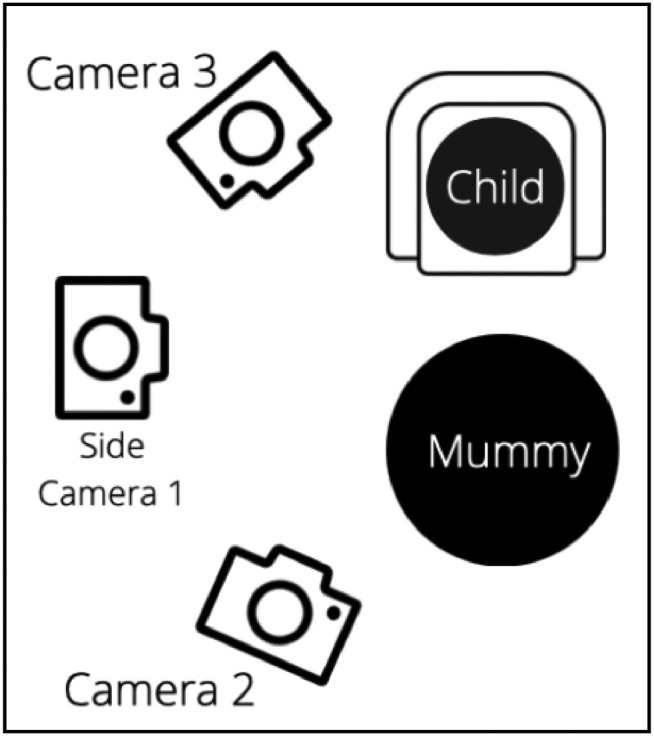
Camera Setup for Part 2.

When transiting from Part 1 to Part 2, experimenters are to clear the presentation tray from the floor. And in place of the presentation tray, the high chair is to be setup and set at a height where the Infant's eye level matches Mother's eye level when he/she is seated in the high chair. Mother is to kneel or be seated in front of the high chair. Camera positions are to capture mainly the play area, infant's torso and face, and mother's face (see Fig. 3 for Part 2 Camera setup).

### Task delivery

#### Part 1 – pre-demonstration free play

E1 should ensure that the toys are correctly arranged on the tray before informing Mother and infant to start the task. The Mother should make a sweeping motion with her hand over the toys while saying “INFANT NAME, let's play with these toys!” to signal the start of the play round. There will be 4 min of free play. After 4 min, the experimenter will give the cue that the task is over by saying, “Okay, end of play time!”. The Mother will then stand up with her infant in her arms and to stand facing a wall to avoid infants observing the setup for Part 2 by the experimenters.

#### Part 2 - Demonstration Phase

The Mother will demonstrate the compressibility of each object one at a time (in random order for each infant). For each object she will say “(infant's name), look!”, then gaze at the object and compress it for 2s using both hands, held sideways. Mothers may repeat or rephrase the prompt according to what feels natural to her. The mother should NOT describe the objects according to their shape or material. E1 will sit behind Mother and pass her the toys in the order they should be demonstrated. The Mother should not turn around to look at the experimenter when receiving a new toy. When performing the compression demonstration, the Mother should not obstruct her eye contact with her infant - compression should be done at the nose level to capture both the Mother's view of her infant and the toy in front. Specifically:

(a) The toy should be angled squarely to meet the infant's eye gaze, not tilted up or down
(b) Ensure that the Mother's fingers are not blocking the compression of toys
(c) Ensure that 2 fingers and a thumb from both hands are used to compress the object
(d) When the Mother has her eye-tracker glasses on, the experimenters should take care to remain out of view to avoid the glasses tracking the experimenter's faces instead of the infant's face.

#### Part 3 – post-demonstration free play

As before, the Mother should make a sweeping motion with her hand over the toys while saying “INFANT NAME, let's play with these toys!” to signal the start of play. There will be 4 min of free play. After 4 min, the experimenter will give the cue that the task is over by saying, “Okay, end of play time!”

During Parts 1 and 3, some infants who are more active might walk away from the toys, experimenters can invite the mother to help bring her infant back to the front of the setup or the experimenters can help bring him/her back to the front of the setup. During the task, both Mother and Experimenters are allowed to call the infant's name to get their attention or to encourage them to continue playing with the toys by using the same starting cue “Let's play with these toys”.

## Sociometric video coding

Sociometric coding of infant and parental behaviour (e.g., for gaze and type/quality of play) should be performed on the video data as detailed in the coding scheme (Table 3) below.

**Table 3:** Sociometric video coding scheme.

STT Task Video Coding Legend	
First round of play: Balls VS Cubes 20: Code for entire duration of this round of play Round starts when mother cues for baby to start playing: “NAME, can you play with these toys?” (or related sentences) Round ends when mother cues for baby to stop playing with the toys: “Okay, that's all for this round of play!” (or related sentences) - Stop coding from start of 4 mins regardless of whether they continue to play after time stops	
Code for INTENTIONAL TOUCH (baby looks at toys and touch it) on toys and events Start Time: when the baby's eye gaze is on toy and touches toy End Time: when the baby stops touching toy or touches a new toy 21.1–0.4/22.1–4/23.1–4/24.1–4: Code for actions during intentional touch 25.1–0.4: Code for intentional gaze with or without actions during intentional touch. Intentional - duration (>0.5 s) .1 - Hard Balls .2 - Soft Balls .3 - Hard Cubes .4 - Soft Cubes *Circular/repetitive motion = Random actions to any objects* *i.e. throwing - throwing outwards or at a person while looking straight in the direction of throw* *Non-circular/repetitive motion = Functionality of toys:* *i.e. Balls - rolling or bouncing (dropping or throwing downwards/ infront of them to demonstrate bouncing) and trying to show the function of the balls* *Cubes - stacking and building actions of putting it on each other or lining it up to build a structure*	
20	Balls/Cubes Play
21.1	Physical contact with Hard Ball and no actions (e.g. holding)
21.2	Physical contact with Soft Ball and no actions (e.g. holding)
21.3	Physical contact with Hard Cube and no actions (e.g. holding)
21.4	Physical contact with Soft Cube and no actions (e.g. holding)
22.1	Physical contact with Hard Ball and circular/repetitive action/motion with toy (e.g. banging, shaking, mouthing, throwing)
22.2	Physical contact with Soft Ball and circular/repetitive action/motion with toy (e.g. banging, shaking, mouthing, throwing)
22.3	Physical contact with Hard Cube and circular/repetitive action/motion with toy (e.g. banging, shaking, mouthing, throwing)
22.4	Physical contact with Soft Cube and circular/repetitive action/motion with toy (e.g. banging, shaking, mouthing, throwing)
23.1	Physical contact with Hard Ball and other (non-circular/non-repetitive) action with toy (e.g. moving it, playing with the toy in its functionality)
23.2	Physical contact with Soft Ball and other (non-circular/non-repetitive) action with toy (e.g. moving it, playing with the toy in its functionality)
23.3	Physical contact with Hard Cube and other (non-circular/non-repetitive) action with toy (e.g. moving it, playing with the toy in its functionality)
23.4	Physical contact with Soft Cube and other (non-circular/non-repetitive) action with toy (e.g. moving it, playing with the toy in its functionality)
24.1	Physical contact with Hard Ball and compressing/pressing into the Hard Ball
24.2	Physical contact with Soft Ball and compressing/pressing into the Soft Ball
24.3	Physical contact with Hard Cube and compressing/pressing into the Hard Cube
24.4	Physical contact with Soft Cube and compressing/pressing into the Soft Cube
25.1	Intentional gaze at Hard Ball
25.2	Intentional gaze at Soft Ball
25.3	Intentional gaze at Hard Cube
25.4	Intentional gaze at Soft Cube
80.x Infant Vocalisations for entire 4 mins Code for Infant Vocalisations or meaningful communication Start Time: infant's first frame of vocalisations End Time: infant's last frame of vocalisations	
80.1	Positive Infant Vocalisations (Include both word/vowel-like utterances as well as other vocal productions that are not clearly linguistic in nature (e.g. grunts, cooing, etc.))
80.2	Negative Infant Vocalisations (crying, whining, fussing noises)
81.x Infant Facial expressions for entire 4 mins Start Time: infant's first frame of expression change End Time: infant's last frame of expression change (coding scheme adapted from Neale et al., 2018)	
81.1	Infant Facial Expressions - Positive
81.2	Infant Facial Expressions - Negative
81.3	Infant Facial Expressions - Neutral affect
*MAINLY INFANT VIDEO IS USED TO CODE FOR GAZE AND TOYS*	
2. Demonstration of Compressibility for Hard and Soft Balls and Cubes by mother 90: Code for entire duration of this round of demonstration Round starts when mother cues for baby to start playing: “NAME, look here” (or related sentences) Round ends when mother cues for baby to stop playing with the toys: “Okay, that's all for this round!” (or related sentences)	
91.1 - 0.4: Duration of each toy demonstration Each toy demonstration start time: starts when mother cues for baby to look at her: “NAME, look here” Each toy demonstration end time: ends when mother changes toy mother calls name of baby to catch attention and demonstrates compressibility of each toy (start and end of demonstration for each toy) - End demonstration when toy leaves mother hand (whether toy was taken by child or whether mother releases toy) 92 - 94: Baby's attention during Demonstration Phase Start Time: when the baby's eye gaze is on mother / the toy held by the mother End Time: when the baby's eye gaze leaves mother / toy held by mother Note: 92 and 93 cannot start earlier/later than 91 (Demonstration Phase; 92 and 93 must be within the demonstration Phase)	
90	Demonstration of toy compressibility
90.1	MATERNAL UTTERANCE: Time/Duration when Mother says Look here OR calls the name of infant/nickname for the attention of baby only - What we are interested in is how many times they repeat their baby's name, define the entire period of the mother talking and use as control for duration (does not mean that each name calling is one row, but we capture the whole duration of calling the name)
90.2	MATERNAL INSTRUCTIONS: Time when Mother prompts the child with other instructions besides name and look here (note down what did the mother say during this time in the comment section)
91 Start: When there is verbal cue from mum. End: End time is when mom body shows that she put down the toy/not holding on the toy any more	
91.1	Duration of Hard Ball demonstration from first cue to change of toy
91.2	Duration of Soft Ball demonstration from first cue to change of toy
91.3	Duration of Hard Cube demonstration from first cue to change of toy
91.4	Duration of Soft Cube demonstration from first cue to change of toy
92 Start: When there is verbal cue from mum. If no cue from mum, take infant's start of attention/gaze on the object. Write a comment to state that is based on attention End: End time is when the baby's attention is broken from that toy being demonstrated	
92.1	Looking at the Hard Ball held by mother and saw demonstration of compressibility for the demonstrated toy - cue with “NAME, look here!” and compressing of toy
92.2	Looking at the Soft Ball held by mother and saw demonstration of compressibility for the demonstrated toy - cue with “NAME, look here!” and compressing of toy
92.3	Looking at the Hard Cube held by mother and saw demonstration of compressibility for the demonstrated toy - cue with “NAME, look here!” and compressing of toy
92.4	Looking at the Soft Cube held by mother and saw demonstration of compressibility for the demonstrated toy - cue with “NAME, look here!” and compressing of toy
93 Start: First frame when infant's attention/ gaze not on the object End: Last frame before infant looks back at the object	
93.1	Looking at other places and did not look at the demonstration of compressibility for the demonstrated toy - Hard Ball
93.2	Looking at other places and did not look at the demonstration of compressibility for the demonstrated toy - Soft Ball
93.3	Looking at other places and did not look at the demonstration of compressibility for the demonstrated toy - Hard Cube
93.4	Looking at other places and did not look at the demonstration of compressibility for the demonstrated toy - Soft Cube
94 Start: First frame for start of reaching actions by the infant End: When the baby's attention is broken from that toy being demonstrated	
94.1	Baby reaching the Hard Ball during demonstration / when demonstration end
94.2	Baby reaching the Soft Ball during demonstration / when demonstration end
94.3	Baby reaching the Hard Cube during demonstration / when demonstration end
94.4	Baby reaching the Soft Cube during demonstration / when demonstration end
95 Start: First frame for fingers/hand touching the object End: Last frame for fingers/hand touching the object (next frame should show the infant hands/fingers off the object)	
95.1	Baby touching the Hard Ball during demonstration / when demonstration end
95.2	Baby touching the Soft Ball during demonstration / when demonstration end
95.3	Baby touching the Hard Cube during demonstration / when demonstration end
95.4	Baby touching the Soft Cube during demonstration / when demonstration end
80.x Infant Vocalisations / Facial expressions Code for Infant Vocalisations or meaningful communication Start Time: infant's first frame of vocalisations End Time: infant's last frame of vocalisations	
80.1	Positive Infant Vocalisations (Include both word/vowel-like utterances as well as other vocal productions that are not clearly linguistic in nature (e.g. grunts, cooing, etc.))
80.2	Negative Infant Vocalisations (crying, whining, fussing noises)
81.x Infant Facial expressions Start Time: infant's first frame of expression change End Time: infant's last frame of expression change (coding scheme adapted from Neale et al. 2018)	
81.1	Infant Facial Expressions - Positive
81.2	Infant Facial Expressions - Negative
81.3	Infant Facial Expressions - Neutral affect
*MAINLY MOTHER/EXPERIMENTER VIDEO IS USED TO CODE FOR WHICH TOY IS DEMONSTRATED / INFANT POV FOR INFANT'S GAZE AND ACTIONS*	
Second round of playing: Balls VS Cubes 30: Code for entire duration of this round of play Round starts when mother cues for baby to start playing: “NAME, can you play with these toys?” (or related sentences) Round ends when mother cues for baby to stop playing with the toys: “Okay, that's all for this round of play!” (or related sentences)	
Code for INTENTIONAL TOUCH (baby looks at toys and touch it) on toys and events Start Time: when the baby's eye gaze is on toy and touches toy End Time: when the baby stops touching toy or touches a new toy 31.1–0.4/32.1.4/33.1–0.4/34.1–0.4: Code for actions during intentional touch. 35.1–0.4: Code for intentional gaze with or without actions during intentional touch. Intentional - duration (>0.5 s or 1 s depending on kid's glance)	
30	Balls/Cubes Play
31.1	Physical contact with Hard Ball and no actions (e.g. holding)
31.2	Physical contact with Soft Ball and no actions (e.g. holding)
31.3	Physical contact with Hard Cube and no actions (e.g. holding)
31.4	Physical contact with Soft Cube and no actions (e.g. holding)
32.1	Physical contact with Hard Ball and circular/repetitive action/motion with toy (e.g. banging, shaking, mouthing, throwing)
32.2	Physical contact with Soft Ball and circular/repetitive action/motion with toy (e.g. banging, shaking, mouthing, throwing)
32.3	Physical contact with Hard Cube and circular/repetitive action/motion with toy (e.g. banging, shaking, mouthing, throwing)
32.4	Physical contact with Soft Cube and circular/repetitive action/motion with toy (e.g. banging, shaking, mouthing, throwing)
33.1	Physical contact with Hard Ball and other (non-circular/non-repetitive) action with toy (e.g. moving it, playing with the toy in its functionality)
33.2	Physical contact with Soft Ball and other (non-circular/non-repetitive) action with toy (e.g. moving it, playing with the toy in its functionality)
33.3	Physical contact with Hard Cube and other (non-circular/non-repetitive) action with toy (e.g. moving it, playing with the toy in its functionality)
33.4	Physical contact with Soft Cube and other (non-circular/non-repetitive) action with toy (e.g. moving it, playing with the toy in its functionality)
34.1	Physical contact with Hard Ball and compressing/pressing into the Hard Ball
34.2	Physical contact with Soft Ball and compressing/pressing into the Soft Ball
34.3	Physical contact with Hard Cube and compressing/pressing into the Hard Cube
34.4	Physical contact with Soft Cube and compressing/pressing into the Soft Cube
35.1	Intentional gaze at Hard Ball
35.2	Intentional gaze at Soft Ball
35.3	Intentional gaze at Hard Cube
35.4	Intentional gaze at Soft Cube
*MAINLY INFANT VIDEO IS USED TO CODE FOR GAZE AND TOYS*	
80.x Infant Vocalisations / Facial expressions Code for Infant Vocalisations or meaningful communication Start Time: infant's first frame of vocalisations End Time: infant's last frame of vocalisations	
80.1	Positive Infant Vocalisations (Include both word/vowel-like utterances as well as other vocal productions that are not clearly linguistic in nature (e.g. grunts, cooing, etc.))
80.2	Negative Infant Vocalisations (crying, whining, fussing noises)
81.x Infant Facial expressions Start Time: infant's first frame of expression change End Time: infant's last frame of expression change (coding scheme adapted from Neale et al. 2018)	
81.1	Infant Facial Expressions - Positive
81.2	Infant Facial Expressions - Negative
81.3	Infant Facial Expressions - Neutral affect

## Example of results and analysis

**Fig. 4:**
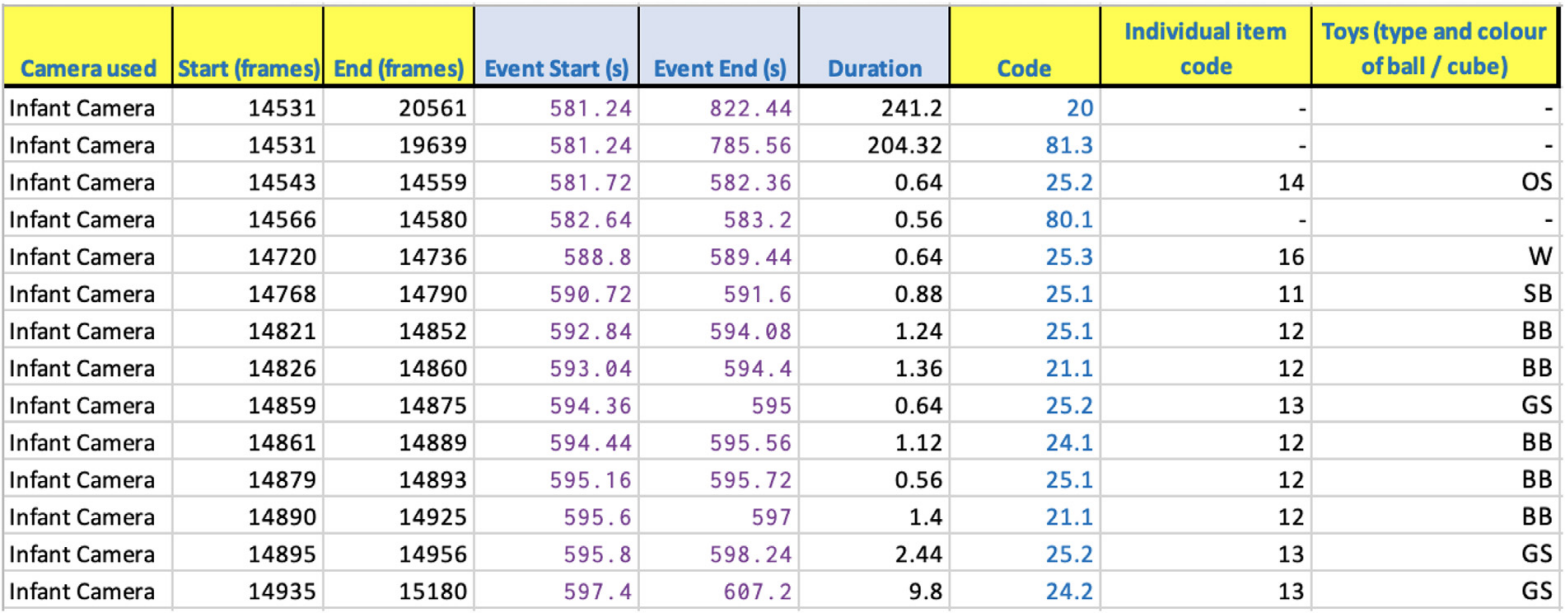
Example of sociometric coding of social behaviours in Part 1.

*Sociometric Coding.* An example of the sociometric coding for social behaviours for an example infant participant is provided in Fig. 4 below.

**Fig. 5:**
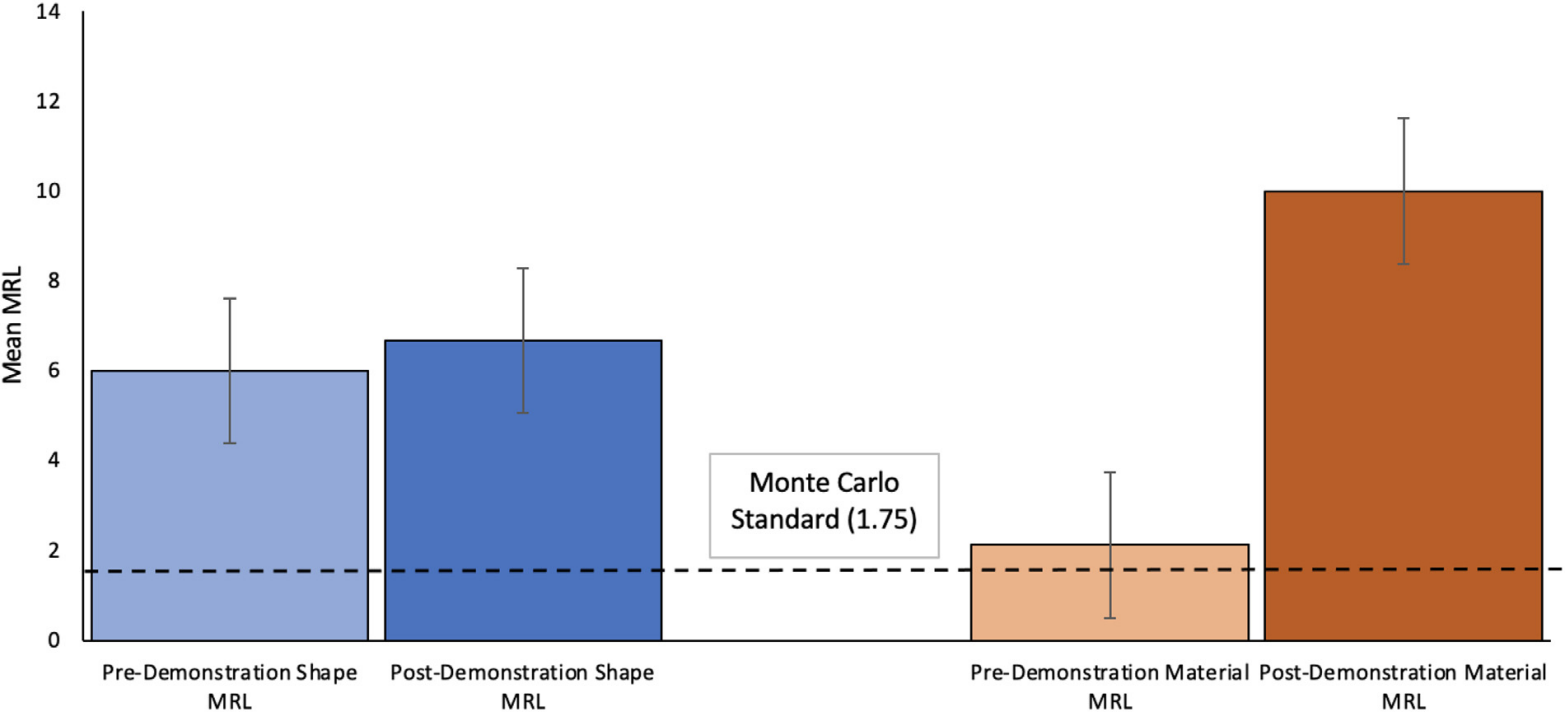
Example of successful shift toward material-based object categorisation from pre- to post- demonstration phases.

*Set-Shifting Performance.* In this task, we are interested in changes in infants’ object categorisation before and after the parental demonstration (i.e. Part 1 to Part 3), as an indication of successful attention set-shifting. To assess object categorisation performance, the classic performance measure - Mean Run Length (MRL) is used. MRLs are calculated using run lengths as the number of touches in a row to objects from the same category (i.e., shape) [2,6]. It is predicted that, comparing performance pre- to post- parental demonstration, infants’ MRLs for *material*-based categorisation will increase, evidencing successful attention shifting toward this dimension. MRLs are calculated by dividing the total number of touches by the total number of runs across all categories, for both the pre- and post-demonstration parts of the task [6]. The calculated MRLs are to be compared against average “random” sequence lengths as produced by a Monte-Carlo simulation to assess performance against chance [2], typically a value of 1.75. Fig. 5 shows an example of a successful attentional shift from shape to material-based object categorisation, as evidenced by increased material MRL.

## CRediT authorship contribution statement

**Xing Xi Tan:** Conceptualization, Methodology, Writing – original draft. **Victoria Leong:** Supervision, Project administration, Writing – review & editing.

## Declaration of Competing Interest

The authors declare that they have no known competing financial interests or personal relationships that could have appeared to influence the work reported in this paper.

## Appendix Supplementary materials

Image, application 1
