# Supplementary material for: A protocol for social interactive assessment of infant attention set-shifting between 12–24 months of age
Source: MethodsX. 2023 Jun 29;11:102273. doi: 10.1016/j.mex.2023.102273 (PMC10336785; doi:10.1016/j.mex.2023.102273)
Supplement: Supplementary file 1 [file mmc1.zip › supp.docx]

**Supplementary material *and/or* additional information [OPTIONAL]**

- - Session sheet and Experimenter Checklist (Supplementary Material Appendix 1)
  - Toy order (Supplementary Material Appendix 2)
  - Parent Instruction sheet (Supplementary Material Appendix 3)
