# Supplementary material for: A protocol for social interactive assessment of infant attention set-shifting between 12–24 months of age
Source: MethodsX. 2023 Jun 29;11:102273. doi: 10.1016/j.mex.2023.102273 (PMC10336785; doi:10.1016/j.mex.2023.102273)
Supplement: Supplementary file 1 [file mmc1.zip › Supplementary Materials.docx]

### **Appendix 1 - Session Sheet And Experimenter Checklist**

PARTICIPANT ID: _________ DATE: __________                E1: __________ E2 ________

ORDER: _________

**Sequential Touching Task Session Sheet**

**PRE-EXPERIMENTAL CHECKLIST**

| **Items** | **Tick after checking** | **Remarks** |
| --- | --- | --- |
| Tray *(check that the raised sides are intact)* |  |  |
| 4 toy blocks *(2 are compressible and 2 are rigid)* |  |  |
| 4 toy balls *(2 are compressible and 2 are rigid)* |  |  |
| Position toys according to order |  |  |
| Video cameras are positioned and charged |  |  |
| Instruction Sheet for Mother |  |  |
| Stopwatch |  |  |
| Toys for distraction |  |  |

**SCORING SHEET (DURING EXPERIMENT)**

| **Task** | **Tick after completion** | **Remarks** |
| --- | --- | --- |
| Part 1 - First Toy Balls vs Toy Blocks  *(4 minutes of free play)* |  |  |
| Part 2 - Demonstration of compressibility |  |  |
| Part 3 - Second Toy Balls vs Toy Blocks  *(4 minutes of free play)* |  |  |

**POST-EXPERIMENT CHECKLIST**

| **Items** | **Tick after checking** | **Remarks** |
| --- | --- | --- |
| Tray *(check that the raised sides are intact)* |  |  |
| All 8 toys are kept *(4 toy blocks, 4 toy balls)* |  |  |
| Clean all toys |  |  |
| Clean the tray |  |  |
| Cameras stopped and connected to power cable |  |  |
| Participant ID and date filled on the session sheet |  |  |

### **Appendix 2 – Toy Order**

Order 1:


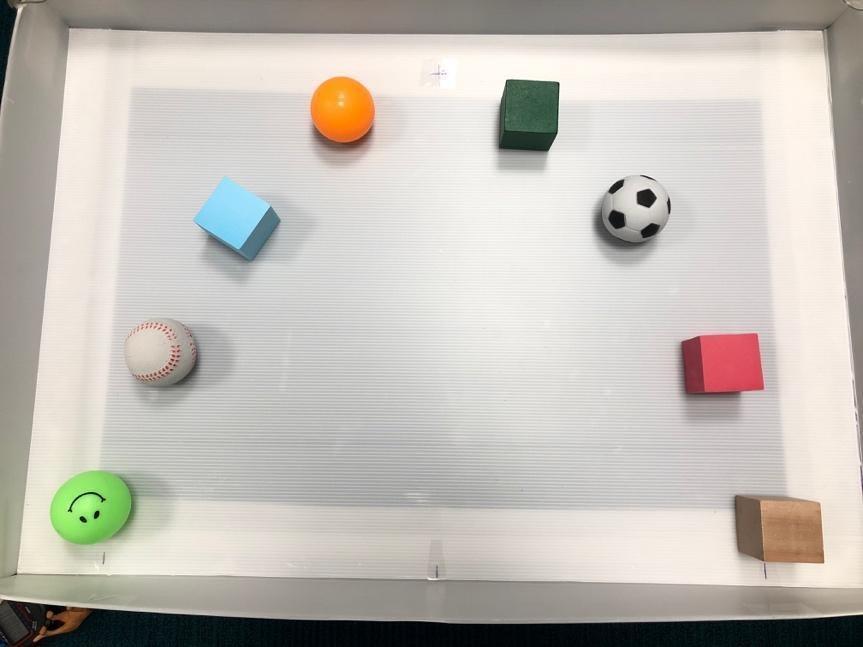


Order 2:


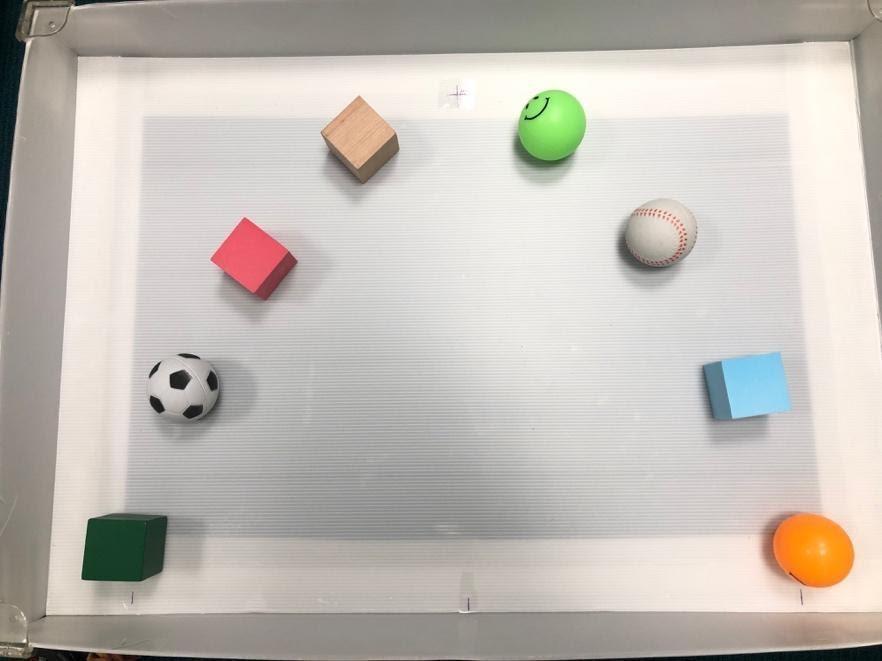


Order 3:


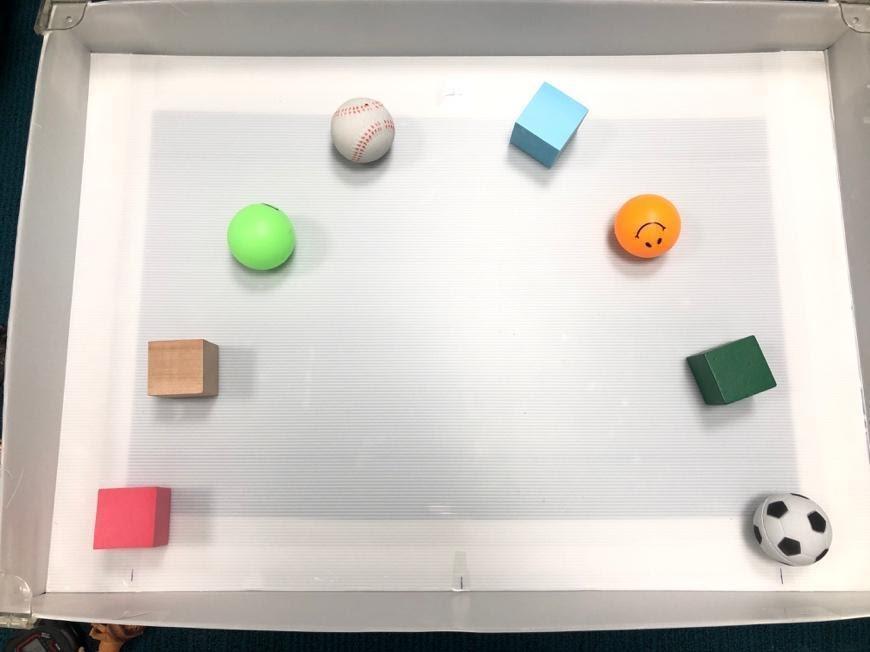


### **Appendix 3 – Parent Instruction Sheet**

Dear Parent, for this task we will require you to be the one interacting with and giving instructions to your child. This instruction sheet is for you to familiarise yourself with the instructions for the task, and we have also attached a video link in our email to view along with this instruction sheet. You can access the [demonstration video (here)](https://drive.google.com/file/d/18ggCiqRjX5e0MnIqeSAs5CiIKuepkT6Y/view?usp=sharing) or via the link that we sent in our email. This demonstration video will show you how the task and setup will look like. If you have any questions, please feel free to contact us!

Part 1

1. Please sit with your child in your lap facing away from the tray while we lay out the objects. We will tell you when to turn around!

2. When you are ready, turn your child around to face the tray with the objects and make a sweeping gesture over the toys whilst saying, **“[CHILD’s NAME], let’s play with these toys!”**

3. Your child will be playing with the toys for 4 minutes. Please remain quiet and allow your child to move towards the objects and play with the objects on his/her own.

4. At the end of 4 minutes, we will give you a signal to tell your child: **“Okay, end of play time!”**

Part 2

1. Please help us to seat your child in the high chair.

2. You will sit opposite your child and squeeze each object for 2 secs to show your child that some objects are soft and some are hard. Please hold each object with both hands, facing toward your child, and squeeze firmly, trying not to tilt the object as you squeeze it.

3. Feel free to call your child’s name to attract his/her attention before squeezing the objects, but please provide NO other comments during this demonstration.

Part 3 (similar to Part 1)

1. Please sit with your child in your lap facing away from the tray while we lay out the objects again. We will tell you when to turn around!

2. When you are ready, turn your child around to face the tray with objects and make a sweeping gesture over the toys whilst saying, **“[CHILD’S NAME], let’s play with these toys!”.**

3. Your child will be playing with the toys for 4 minutes. Please remain quiet and allow your child to with the objects on his/her own.

4. At the end of 4 minutes, we will give you a signal to tell your child: **“Okay, end of play time!”**

*Instructions continue on the next page*
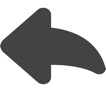


Please note: it is important that you do not influence what objects your child decides to play with throughout this session, therefore, please try to adhere to the following do’s and don'ts:

| **Do** | **Don’t** |
| --- | --- |
| During Part 2, call your child’s name to attract their attention. | Do not name the shape or the material of the toys |
| If your child is distracted, encourage s/he to play with the objects presented. | Do not initiate any interaction with your child, or with the toys during the play time in Parts 1 & 3. |
| If necessary, translate the prompts to your native language. |  |
| If your child places the toy in his/her mouth, gently pull their hand away. |  |
| If your child throws the toys out of the tray or hands a toy to you, take the object quietly with a neutral facial expression and hand it to one of the experimenters when your child is not looking in your direction. |  |
